# Editorial: Crosstalk in ferroptosis, immunity & inflammation

**DOI:** 10.3389/fimmu.2025.1587075

**Published:** 2025-04-03

**Authors:** Yu’e Liu, Guangzhen Wu, Linjing Feng, Qi Feng, Hua Zhong

**Affiliations:** ^1^ Department of Neurosurgery, Shanghai East Hospital, School of Medicine, Tongji University, Shanghai, China; ^2^ Boston Children’s Hospital, Harvard Medical School, Boston, MA, United States; ^3^ Shanghai Fourth People’s Hospital, School of Medicine, Tongji University, Shanghai, China; ^4^ Department of Oncology Surgery, Shanghai Mengchao Cancer Hospital, Shanghai University, Shanghai, China; ^5^ Research Institute of Nephrology, Zhengzhou University, the First Affiliated Hospital of Zhengzhou University, Zhengzhou, China; ^6^ Cancer Epidemiology Division, Population Sciences in the Pacific Program, University of Hawaii Cancer Center, University of Hawaii at Manoa, Honolulu, HI, United States

**Keywords:** ferroptosis, immunity, inflammation, cancer, other diseases

Ferroptosis, an iron-dependent form of regulated cell death characterized by iron overload and lipid peroxidation, has gained significant attention because of its involvement in various pathological conditions, including cancer, neurodegenerative diseases, and inflammatory disorders (1, 2). The intricate interplay between ferroptosis, immune responses, and inflammatory pathways remains a key area of research, with implications for therapeutic strategies targeting these mechanisms. This Research Topic, “*Crosstalk in Ferroptosis, Immunity & Inflammation*,” presents a collection of 24 articles that explore the multifaceted roles of ferroptosis across various disease contexts. By examining how ferroptotic processes intersect with immune responses and inflammatory pathways, these studies shed light on potential mechanisms underpinning a broad spectrum of conditions—from inflammatory and autoimmune disorders to cancer, neurodegeneration, metabolic diseases, and organ-specific injuries.

## Ferroptosis in inflammatory and autoimmune diseases

Paper in this Research Topic discuss the pivotal role of ferroptosis in the pathogenesis of inflammatory diseases such as inflammatory bowel disease (IBD) (Xie et al.), systemic lupus erythematosus (SLE) (Zhao et al.), and lupus nephritis (Fan et al.). Iron metabolism dysregulation and oxidative stress-induced cell death contribute significantly to these pathologies. Investigations into immune imbalances, particularly T-cell-mediated responses, underscore the link between ferroptotic pathways and chronic inflammation in autoimmune disorders. Understanding these mechanisms offers potential therapeutic strategies to mitigate disease progression by targeting ferroptosis-related pathways. Furthermore, oxidative stress and lipid peroxidation—hallmarks of ferroptosis—have been shown to amplify inflammatory responses in chronic diseases (3). The delicate balance between ferroptotic cell death and immune regulation is crucial for maintaining tissue homeostasis. Research suggests that inhibiting ferroptosis may protect against excessive inflammation, while the controlled induction of ferroptosis could be leveraged to eliminate hyperactive immune cells that contribute to autoimmune diseases (4).

## Ferroptosis in cancer and tumor immunology

Ferroptosis is increasingly recognized as a key factor in tumor biology (5, 6). Xia et al. have implicated ferroptosis in cancers, including colorectal cancer, hepatocellular carcinoma (Yang et al.), and lung cancer (Gao and Zhang). Regulators such as SLC7A11 in ferroptosis emerges as promising targets for tumor therapy (7) (Jiang and Sun). Ferroptosis appears to synergize with immunotherapy to enhance antitumor responses, offering new perspectives for cancer treatment. The emerging role of long noncoding RNAs (lncRNAs) in regulating ferroptosis sheds light on novel molecular interactions influencing tumor progression (Chen et al.). Additionally, ferroptosis-based therapies may overcome resistance to traditional chemotherapies and improve patient outcomes by modulating the tumor microenvironment. One of the key mechanisms by which ferroptosis influences cancer progression is through its impact on immune cell infiltration and tumor-associated macrophages. Moreover, the release of damage-associated molecular patterns (DAMPs) from ferroptotic tumor cells can activate antigen-presenting cells, thereby modulating adaptive immune response. Harnessing ferroptosis offers exciting avenues for overcoming chemoresistance and improving the efficacy of immune checkpoint inhibitors.

## Ferroptosis in neurodegenerative and neurological disorders

Ferroptosis has emerged as a key player in neurodegenerative diseases, contributing to neuronal damage through iron accumulation and oxidative stress. In Alzheimer’s disease and Parkinson’s diseases, iron accumulation and lipid peroxidation contribute to neuronal loss and cognitive decline. Additionally, ferroptosis is involved in neuroinflammation by modulating microglial activation, which in turn influences neuronal survival and disease progression. Emerging therapeutic strategies—ranging from antioxidant-based approaches to iron chelation—are being explored to protect neuronal integrity by targeting ferroptotic pathways. In addition to classical neurodegenerative disorders, ferroptosis also plays a critical role in sepsis-associated encephalopathy (SAE), a severe neurological complication of sepsis. Microglia-derived CXCL2 has been shown to promote neuronal ferroptosis in SAE by activating Jun, exacerbating cognitive dysfunction and neuronal loss. Inhibiting CXCL2 or Jun has demonstrated neuroprotective effects, suggesting that targeting ferroptosis could be a novel therapeutic strategy for mitigating SAE-induced brain injury (Yang et al.).

## Ferroptosis in metabolic, renal, pulmonary, and cardiovascular diseases

Chronic metabolic disorders, including diabetes and obesity, are often accompanied by ferroptosis-driven tissue damage. Hyperglycemia-induced oxidative stress can lead to ferroptotic cell death in renal and vascular tissues, contributing to diabetic nephropathy and atherosclerosis. Understanding the metabolic regulation of ferroptosis could help in designing effective interventions for metabolic syndrome and related disorders (8). Additionally. Liu et al. have identified key ferroptosis-related genes involved in endometriosis pathogenesis, highlighting the potential for ferroptosis-targeting therapies to mitigate disease progression and immune dysfunction.

Kidney diseases is a typical metabolic disorder. Dysregulation of iron homeostasis during ferroptotic processes significantly influences progression of kidney diseases, including acute kidney injury and renal fibrosis (9, 10) (Long et al.). While alterations in lipid metabolism also modulate ferroptotic cell death. These findings highlight the need for further mechanistic studies to fully understand these pathways. In renal ischemia–reperfusion (I/R) injury, ferroptosis contributes to kidney damage, which is characterized by increased collagen deposition, α-SMA expression, and oxidative stress. Fer-1 treatment or ATF3 knockdown alleviated these changes while modulating Nrf2/HO-1 signaling. Additionally, exosomal miR-1306-5p release under hypoxia-reoxygenation promoted M2 macrophage polarization, linking ferroptosis to immune regulation in renal fibrosis (Tang et al.). Moreover, Li et al. ‘s study investigating the role of ferroptosis in acute kidney injury (AKI) revealed that GSTT1 and GSTM1 were significantly downregulated in the renal tubular cells of AKI patients and in cisplatin-treated mice. Notably, Gstm1/Gstt1 double knockout (DKO) mice, while phenotypically normal under baseline conditions, presented exacerbated kidney dysfunction, increased ROS levels, and severe ferroptosis following cisplatin administration, underscoring the contribution of GST enzymes to ferroptotic resistance in renal injury.

In chronic obstructive pulmonary disease (COPD), ferroptosis-mediated alveolar epithelial cell death exacerbates disease progression. Exposure to electronic nicotine delivery systems (ENDS) has been linked to worsening COPD features, including emphysema, mucus accumulation, inflammation, and fibrosis, all of which are associated with lipid dysregulation and ferroptotic injury(Han et al.). Beyond pulmonary diseases, ferroptosis also plays a crucial role in I/R injury, a pathological process affecting multiple organ systems, including the heart, brain, lung and kidneys. Ferroptotic cell death contributes to endothelial dysfunction, which in turn promotes vascular inflammation and atherosclerosis. In cardiovascular conditions such as heart failure and myocardial infarction, oxidative stress-induced ferroptosis of cardiomyocytes accelerates disease progression, particularly in cases involving iron overload. Additionally, ferroptosis has been implicated in cerebral I/R injury, where it exacerbates neuronal damage and neuroinflammation (He et al.), further linking ferroptotic mechanisms to both cardiovascular and neurological pathologies. Given its broad impact, targeting ferroptosis represents a promising therapeutic strategy for mitigating I/R injury, reducing vascular complications, and improving outcomes in cardiopulmonary diseases.

## Emerging perspectives and future directions

The rapid evolution of ferroptosis research has opened new therapeutic frontiers across various medical disciplines. Studies exploring anastasis—the recovery from ferroptotic stress—could unveil novel mechanisms of cellular resilience. Advanced bioinformatics analyses have identified key molecular signatures linking ferroptosis with immune infiltration, further reinforcing the importance of computational approaches in this field. Looking ahead, the development of reliable biomarkers, targeted inhibitors, and inducers of ferroptosis will be crucial. Moreover, integrating ferroptosis modulation with immunotherapy holds significant promise for clinical translation across a range of conditions, particularly in oncology and inflammatory diseases.

## Conclusion

This Research Topic provides valuable insights into the crosstalk between ferroptosis, immunity, and inflammation across diverse disease models. By understanding its molecular mechanisms and interactions with immune responses, inflammation, and metabolic pathways, we can identify novel therapeutic targets and improve treatment strategies for a wide range of conditions. Future research should focus on ferroptosis-modulating strategies to enhance therapeutic efficacy, minimize disease progression, and improve patient outcomes. The findings presented in this Research Topic pave the way for future investigations and potential clinical applications, emphasizing the critical role of ferroptosis in immune regulation and disease pathogenesis.
